# Historical Biogeography of Earwigs

**DOI:** 10.3390/biology11121794

**Published:** 2022-12-09

**Authors:** Simone Fattorini

**Affiliations:** Department of Life, Health and Environmental Sciences, University of L’Aquila, Via Vetoio, 67100 L’Aquila, Italy; simone.fattorini@univaq.it

**Keywords:** Cladistic Analysis of Distributions and Endemism, Dermaptera, dispersal, endemism, Gondwana, historical biogeography, Pangea, vicariance

## Abstract

**Simple Summary:**

Earwigs (Dermaptera) have their highest diversity in the tropical areas of the southern hemisphere, whereas the temperate regions of the northern hemisphere have relatively few species. This pattern has been considered a reflection of their origin in Gondwana, the supercontinent that grouped most of the land masses in today’s southern hemisphere, including Antarctica, South America, Africa and Madagascar. An analysis of the distributions of the major evolutionary lineages of earwigs supports the role of the Gondwanan breakup in determining the current patterns of their diversity, as well the influence of the Indian collision with the Eurasian plate. The dispersal into the Eurasian plate was largely constrained by the Himalayan orogenesis and the presence of colder temperatures. This climatic barrier was probably the most important factor that largely hampered the colonization of North America from South America.

**Abstract:**

The Dermaptera are an insect order exhibiting their highest diversity in the tropical areas of the southern hemisphere. This pattern has been considered a reflection of a Gondwanan origin. However, this hypothesis has not been tested through analytical methods. In this paper, the world distribution of earwigs was analysed by using the ‘Cladistic Analysis of Distributions and Endemism’ (CADE), a method which groups areas of endemism on the basis of shared distributions and phylogenetic relationships among taxa. In addition, clustering techniques were applied to depict biotic relationships based on similarity indices. Results of CADE support the idea that Gondwanan fragmentation exerted a crucial role in shaping the current distribution of the main clades of earwigs. However, the relationships between India with South East Asia suggested a biotic interchange occurred after the Indian collision with the Eurasian plate. The overall scenario emerging from cluster analyses revealed a strong influence of dispersal events. Overall, the distribution of earwig major clades indicates that their biogeographical history was mainly characterized by vicariance events (led by the break-up of Gondwana) followed by large scale dispersal processes constrained by the Himalayan orogenesis and the presence of colder temperatures, which have largely hampered the colonization of the northern hemisphere.

## 1. Introduction

The Dermaptera (commonly known as earwigs) are a small group (some 2000 species worldwide [1]) of hemimetabolous insects belonging to the Polyneoptera clade [2]. According to recent phylogenetic reconstructions, Dermaptera include two clades: one corresponding to the extinct suborder Archydermaptera and the other grouping the extinct “Eodermaptera” (which are considered paraphyletic) and the Neodermaptera (which include the formerly widely accepted suborders Forficulina, Hemimerina, and Arixeniina) [3]. Some authors suggest that the Dermaptera probably originated in the Middle Jurassic, but others suppose they probably originated during the early Mesozoic [3]. Neodermapterans first appeared in the Early Cretaceous but might have originated in the Late Jurassic [3].

Dermaptera are mainly associated with warm and humid climates, thus exhibiting their highest diversity in the tropical areas of the southern hemisphere, whereas the temperate regions of the northern hemisphere have fewer species [4]. Because of this concentration of species in the southern hemisphere, the Dermaptera were among the first organisms whose distribution was considered a reflection of vicariance events following Gondwanan fragmentation [5,6,7]. Quite surprisingly, however, this hypothesis has not really been tested analytically and remained at the stage of a narrative interpretation.

Ball [8] suggested that biogeographical research develops through three stages: descriptive, narrative, and analytical, and the evolution of our knowledge of earwig biogeography may be considered exemplificative of them. In the descriptive stage, the complexity of current distributions is recognized and described, but little attempt is made to explain their historical causes. In the case of Dermaptera, the descriptive stage has culminated, at global level, in the publication of early attempts to provide global sketches of their distribution [9,10,11,12]. In the narrative stage, present distributions are recognized as patterns, and historical factors are evoked to explain them. These explanations are developed by inductive observations of the distribution patterns, and they can be rational, but only in a retrospective manner, and thus cannot be tested. At regional level, exemplary of this stage is Bey-Bienko’s [13] treatment of the Dermaptera of URSS, which recognizes, in the Palearctic region, two main centers of differentiation: one in the Mediterranean Basin; the other in an eastern region encompassing the Ussuri basin, Northern China, Japan, Korea, Manchuria and Tibet, and thus corresponding to the “Paleoarchearctic” region of Semenov Tian-Shanskij [14]. At global level, Popham’s explanations of earwig distributional patterns using evolution of Earth’s landmasses are an example of this stage [5,6,7]. In the analytical stage, propositions are explicitly formulated a priori and then tested. Although analytical cues can be found in some of the aforementioned works (especially [7,10]), there is no recent attempt at elucidating the biogeographical history of Dermaptera in an analytical framework.

Many methods have been proposed to reconstruct area-relationships in historical biogeography [15,16]. In general, the best evidence for area-relationships should come from congruent phylogenetic patterns among multiple, unrelated taxa [17]. However, detailed phylogenetic reconstructions are often lacking, especially when large clades are considered. In these cases, heuristic alternatives have been proposed [16]. In this paper, we applied a method called ‘Cladistic Analysis of Distributions and Endemism’ (CADE), which groups the areas on the basis of shared distributions according to parsimony principles and which incorporates cladistic information by coding distributions for more inclusive hierarchical levels [18,19,20,21,22,23,24,25,26,27,28,29].

We applied CADE to the world extant Dermaptera to test if the present distribution patterns can be explained by vicariance events led by major steps in Earth’s history. In addition, we used clustering techniques to depict biotic relationships based on similarity indices.

## 2. Materials and Methods

### 2.1. Areas of Endemism

**Figure 1:**
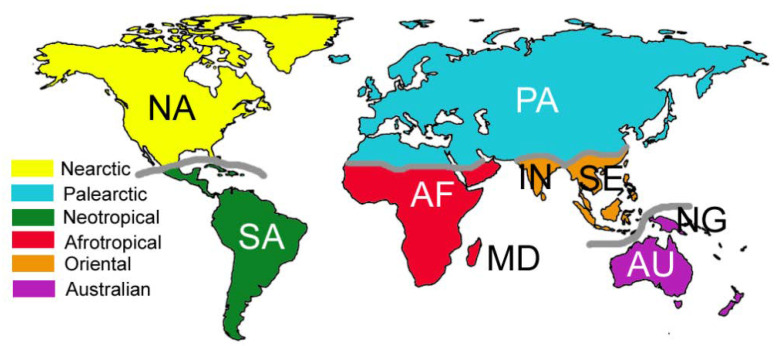
Zoogeographical regions and areas of endemism used in this study. Areas of endemism: AF = Arica (south of the Sahara), AU = Australia, IN = India, MD = Madagascar, NA: North America, NG = New Guinea, PA= Palearctic, SA = South America, SE = South East Asia.

In historical biogeography, the fundamental units of the analysis are called “areas of endemism” [16]. The areas of endemism utilized in this paper are based on Wallace’s zoogeographical regions [30] since data on taxon distributions are currently available mostly according to this scheme [7,12]. However, some internal subdivisions were introduced to better reflect Earth’s history (Figure 1). Namely, the Oriental region was divided into an area corresponding to the Indian subcontinent and an area corresponding to South East Asia, as India was originally part of Gondwana. Madagascar and New Guinea were considered separately from the rest of the Afrotropical and the Australian regions, respectively, because of their insular character. Because earwigs are virtually absent in Greenland, their distribution in the Nearctic region coincides with their presence in North America north of Mexico. South America is used in a broad sense, including a large part of Mesoamerica, thus corresponding to the Neotropical region, because earwig clades occurring in Mesoamerica have their distribution (or are offshoots of clades) centered in South America. With Africa we mean the sub-Saharan part of the continent, which corresponds to the Afrotropical region, as earwigs are absent in the Sahara. The boundary between the Oriental and the Australian regions was set in correspondence with Lydekker’s line [30] because the latter best marks the limit of earwigs distributed in the Oriental region [7]. For this study, raw distributional data were extracted from Popham [7], who outlined the world distribution of major earwig lineages, for all taxa which are considered likely monophyletic, with the addition of Arixiidae and Hemimeridae, which were non-considered by Popham [7]. These data were complemented or refined with further information retrieved from Brindle [31] for Madagascar; Haas [32] and Stuart et al. [33] for Australia; and Srivastava [34,35,36] for the Oriental region.

### 2.2. Taxa

As a whole, a total of 66 original clades (corresponding to genera, subfamilies, or families, depending on the resolution adopted by Popham [7] to code distributional information) were considered. We are aware that this information is inhomogeneous in terms of taxonomic resolution, but a homogeneous finer classification would be even more problematic. The use of species or genera, in most cases, would be of scarce utility, as it would only inflate the number of taxa endemic to single areas, and non-shared taxa are not biogeographically informative of area-relationships. Thus, in most cases, we referred to subfamilies, or genera when they represent highly differentiated clades, as done by Popham [7].

A presence/absence (0 = absence, 1 = presence) matrix was compiled to code distributional information, with each row corresponding to a taxon, and each column to an area of endemism, as defined above (Table S1). Although absences may include both true absences (a taxon is really absent from a certain area) and false absences (the taxon is present, but not recorded), the relatively well-known distribution of Dermaptera and the use of broad areas for geographic coding make it improbable that zeros are due to the lack of records.

To incorporate phylogenetic information into the original taxon per area matrix as requested by CADE (see below), we referred to the most recent available phylogenetic reconstructions [37] and current taxonomic arrangements [1,38,39] (Figure S1). Thus, available cladistic/taxonomic information was used to group genera into subfamilies or families, and subfamilies into families, when recognized as monophyletic; similarly, families and unnamed lineages were further grouped into larger monophyletic groups following the earwig phylogeny as expressed by the cladogram presented by Wipfler et al. [37] complemented with taxonomic information for clades not included in their analyses. The family Haplodiplatyidae was considered in the data set (Table S2), but it is not represented in the phylogenetic reconstruction of Figure S1 because of its unclear relationships [39,40].

### 2.3. Data Analysis

Rosen [41] introduced the Parsimony Analysis of Endemicity (PAE) to identify areas with shared presence of taxa using a cladistic approach. In PAE, areas are treated as taxa and taxa are treated as characters. Thus, areas are grouped by their shared taxa according to the most parsimonious cladogram(s). Taxa occurring in more than one area are useful to reconstruct area-relationships, while those found in a single area are not informative of area-relationships (being equivalent to autapomorphies), but can be useful to identify areas which host, for example, large numbers of endemics. Cladistic Analysis of Distributions and Endemism (CADE) was originally proposed by Cracraft [42] as an alternative to PAE, and later called CADE by Porzecanski and Cracraft [18], to incorporate cladistic information by coding distributions for more inclusive hierarchical levels. Both PAE and CADE infer relationships among the areas of endemism and from that inference extinction and dispersal can be interpreted. However, CADE differs from PAE in the use of both areas and taxa. Differently from PAE, which can be applied to any type of area for the most diverse purposes (including the discovery of areas of endemism), CADE has been specifically conceived as a method to extract historical relationships among a priori established areas of endemism, which are predetermined on the basis of congruence of distributions across the target taxa [16]. As regards the taxa, unlike PAE, CADE incorporates cladistic information into the data matrix by coding distributions of higher hierarchical levels. Thus, even though the detailed relationships among the taxa within the given group to which they belong are not fully resolved by current taxonomic arrangements, the patterns of area relationships implied by their more recent common ancestry relative to taxa in other groups are incorporated into the analysis by coding the hierarchical level above them.

The presence/absence matrix reported in Table S2 was then analysed by maximum parsimony using the software PAUP* (version 4.0a169 [43]). We searched for the most-parsimonious cladogram(s) by using the branch-and-bound searching procedure with furthest addition sequence and ‘MulTrees’ options. All characters were coded as unweighted. An all-zero hypothetical area was used to root the cladogram [18]. Reconstruction of character (taxon distribution) states was optimized using either accelerated or delayed transformation (ACCTRAN and DELTRAN, respectively) [44] ACCTRAN gives a preference for a single origin followed by reversal, so that secondary losses of a taxon are made more likely than independent colonization, whereas DELTRAN gives a preference for two origins of a character state (parallelism). Thus, in PAE and CADE, DELTRAN leads to a preference for dispersal, whereas ACCTRAN for vicariance [20].

Consistency index (ci), retention index (ri), rescaled consistency index (rc) and homoplasy index (hi) were computed to evaluate how well individual characters fit on a phylogenetic tree. If a character has no homoplasy, ci = 0. By contrast, ri = 0 when a character fits the tree as poorly as possible. To express how well the tree described a data set, the overall consistency index (CI, which equals one when there is no homoplasy and decreases as homoplasy increases), the retention index (RI, which expresses the amount of synapomorphies in a data set) and the rescaled consistency index (RC, which is the product of CI and RI) were calculated. Data robustness was evaluated with 10,000 bootstrap replicates, each with a single heuristic search using the following default options: (1) starting trees obtained via stepwise addition, with one tree held at each step, (2) simple addition sequence (reference area was the outgroup), (3) tree-bisection-reconnection, and (4) ‘MulTrees’ option in effect. The use of bootstrapping to assess confidence limits on phylogenies has been questioned [45], and bootstrap values were used here only as a valuable estimate of the robustness of a data set [46,47].

In addition to CADE, we used cluster analysis procedures to highlight biotic relationships among areas of endemism. Cluster analyses of biogeographical similarities have been proposed as a tool to investigate the influence of current and historical factors in species distributions (e.g., [48,49,50,51,52,53,54,55,56,57,58]). CADE and cluster analyses are philosophically, conceptually, and operationally vastly different methods. While CADE should only reflect historical relationships (mainly assumed to be the result of vicariance events), cluster analyses are phenetic approaches aimed at disclosing similarities, which may arise from different causes. Thus, in a holistic approach, the two methods may reciprocally illuminate area-relationships. We applied cluster analyses to the data of Table S1 with two dissimilarity coefficients commonly used to depict biogeographic relationships: the Dice-Sørensen coefficient (βsor), to express overall dissimilarity, and the Simpson coefficient to highlight the pure turnover. βsor is calculated as:

βsor = (b + c)/(2a + b + c),
(1)

where a is the number of species present in both areas, b is the number of species present in the first area, but not in the second, and c is the number of species present in the second area, but not in the first. This coefficient is one of the most used measures in biogeography due to its dependence on the proportion of species shared between two communities, but it has the potential disadvantage of incorporating both true spatial turnover and differences in richness [59]. βsim is calculated as:

βsim = min(b,c)/(a + min(b,c)),
(2)

and expresses the compositional turnover among areas independently from richness gradients [60,61,62,63,64]. It is therefore used to highlight the pure turnover patterns.

Both these coefficients are not biased by species absences; i.e., the similarity between areas A and B is independent of area C. In both cases, dissimilarity coefficients were clustered using the UPGMA (unweighted pair-group method with arithmetic average) amalgamation rule rather than single-linkage (nearest neighbor in a cluster) or complete-linkage (furthest neighbor) algorithms because it is considered the clustering strategy that minimizes the distortion of the original data matrix [52]; therefore, it is favored particularly in biogeographical studies (e.g., [48,49,52,65,66,67]).

Since dendrogram topology and bootstrap supports of cluster analyses are affected by the order of areas in the original matrix (especially when pairwise distance values are equal) [68,69], we used a procedure which re-samples the order in which areas are included in the analyses and creates consensus trees in bootstrap analysis [68,69]. For this purpose, the package recluster [70] was used with R 4.0.2 [71]. We evaluated the impact of alternative topologies with the function recluster.node.strength, which (1) compares the initial tree with consensus trees obtained among trees (n = 1000) produced after re-ordering of sites for six different consensus rules (from 0.5 to 1 with step 0.1), and (2) reports the percentage of times each node is repeated among different consensus rules [68,72]. Then, a bootstrap analysis was applied to the 0.5 consensus tree with the function ‘recluster.boot’ (tree = 1000, boot = 1000, level = 1).

## 3. Results

**Figure 2:**
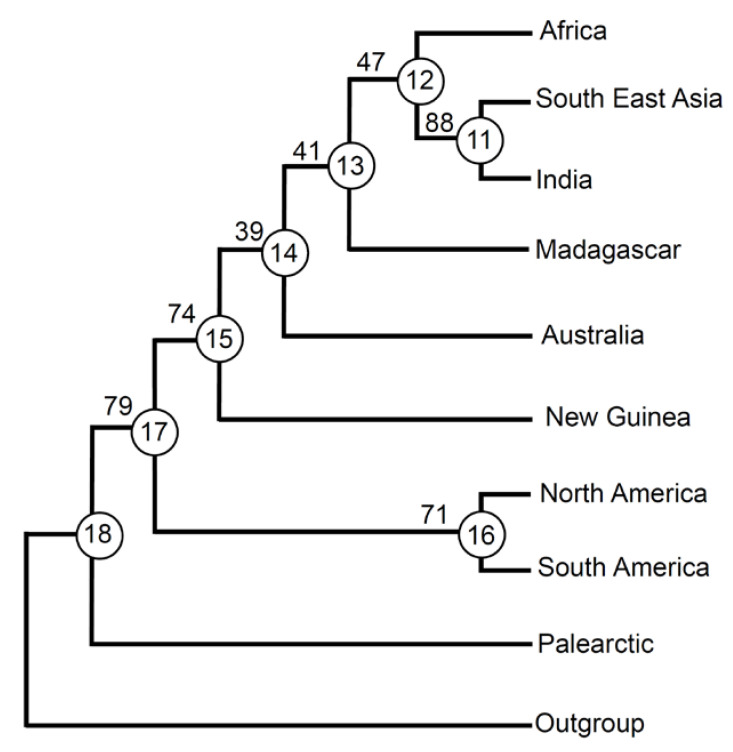
Area relationships found by Cladistic Analysis of Distributions and Endemism (CADE). Circled numbers indicate nodes. Numbers on branches are bootstrap values (percentages).

The analysis resulted in only one most parsimonious tree (Figure 2), with 130 steps. Values of CI excluding uninformative characters (0.505), RI (0.535), and RC (0.341) indicate a relatively low level of homoplasy, which suggests that the vicariance signal is rather strong in the data set.

Although not all branches are well supported by bootstrap values, the tree is completely resolved. For the 48 informative taxa, 11 have ci = 1 and ri = 1 (hi = 0) (Table S3). This indicates that no extinction/dispersal events affected their distributions, which can be entirely attributed to vicariance. These taxa (present in all areas of a clade and only here) also represent unambiguous apomorphies among areas. Apomorphies with ci = 1 are found in all nodes and terminal areas, with the following exceptions: node 15 → node 14 and node 15 → New Guinea, node 13 → 12, and node 16 → North America (Tables S3 and S4).

The fact that New Guinea and North America have only apomorphies with ci = 0.500 or 0.333 indicates that the distribution of the taxa supporting these nodes cannot be explained by vicariance alone but were affected also by extinction/dispersal.

Examination of character changes revealed 83 unambiguous 0→1 changes, and 8 unambiguous 1→0 changes (Table S4). Under ACCTRAN assumptions, there were 13 ambiguous 0→1 changes and 13 ambiguous 1→0 changes; by contrast, under DELTRAN assumptions, there were 24 ambiguous 0→1 changes and 2 ambiguous 1→0 changes (Table S4). This means that, as expected, DELTRAN increases the possibilities that different areas obtained the same taxon independently (via dispersal). This was particularly relevant for node 12 → Africa (with six 0→1 ambiguous changes in addition to the seven unambiguous 0→1 changes also occurring in ACCTRAN) and for node 14 → Australia (with three 0→1 ambiguous changes in addition to the three unambiguous 0→1 changes also occurring in ACCTRAN; note that for these steps, only unambiguous 0→1 changes occur in ACCTRAN).

Palearctic appears as the sister to all other areas. The second, most basal sister relationship is between South and North America and all remaining areas. Within this latter branch, the most basal position is represented by New Guinea. Then, Australia is separated from all others, which include areas belonging to the Afrotropical and Oriental regions. Madagascar occupies the most basal position, whereas Africa is the sister to South East Asia and India.

**Figure 3:**
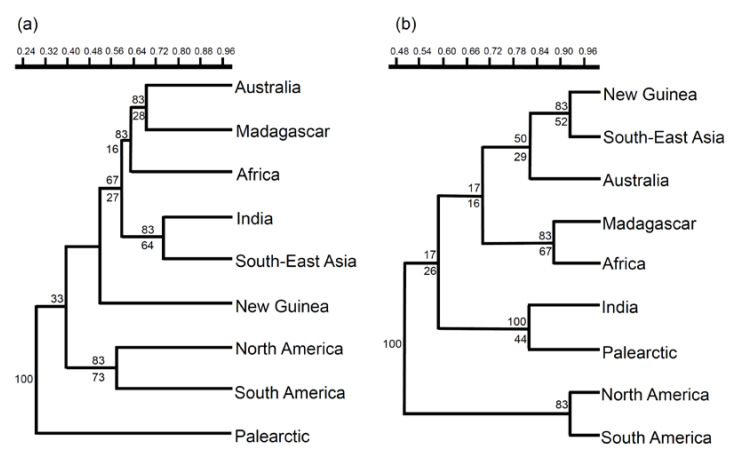
Cluster analysis (UPGMA) of areas of endemism based on Dice-Sørensen (**a**) and Simpson (**b**) coefficients. Numbers on branches are the percentages of times each node was repeated among different consensus rules. Numbers below branches are bootstrap values (percentages) for the consensus tree obtained after 1000 permutations of the order in which areas are analysed.

The use of cluster analysis with Dice-Sørensen coefficient (βsor; Figure 3) produced unstable results, which are however substantially compatible with those obtained with CADE. The most striking difference is, however, the position of Australia, which is potentially associated with Madagascar. When only the turnover component is considered (using the Simpson βsim coefficient; Figure 3), the resulting tree is more supported and shows a clear separation of the Americas from all other areas; the Palearctic is clustered with India, while Madagascar has a strong affinity with Africa; New Guinea is clustered with South East Asia.

## 4. Discussion

Before the proposal of plate tectonic theory, the hypothesis of land mobility was not taken seriously by most geologists and Alfred Wegener’s theory of continental drift remained substantially ignored [30,73,74]. The enormous potential of the continental drift theory for the explanation of current distributional patterns was ignored also by most biologists, with the notable exception of René Jeannel [75], who found in the continental drift the rationale for a plausible explanation of current distributions for a multitude of organisms. Jeannel’s work, however, was largely ignored or ridiculed (see, for example, the position expressed by Darlington [76] and Rehn [77]) and the hypothesis of continental drift was regarded as unproved or useless [78]. It is emblematic of this general skepticism the position of the author of an introductory textbook, who, although inclined to consider seriously the enormous impact that the theory of continental drift could have had in biogeography, concluded that there was “no conclusive reason why the theory of the permanence of the continents should be rejected” [79]. With the increasing acceptance of land mobility in geology in the early 1960s, Dermaptera offered one of the first examples of current distribution patterns that could be interpreted as a result of movements of large parts of the Earth’s crust [5,6,7]. However, despite the recent progresses in historical biogeography [16,30,71], the historical biogeography of earwigs has not been tested with modern techniques.

In this paper, the use of the Cladistic Analysis of Distributions and Endemism (CADE, a method which groups areas of endemism on the basis of shared distributions and phylogenetic relationships among taxa) supported the idea that Gondwanan fragmentation exerted a crucial role in shaping the current distribution of the main clades of earwigs, although complex dispersal events have largely contributed to current earwig distributions.

Before interpreting biologically the results of CADE it is important to consider the effects that the small number of species and the high incidence of endemics may have on the recovered patterns [20]: (1) since the outgroup is an all-zero area, areas having few species will tend to be viewed unduly as being primitive, and (2) high levels of endemism obviously diminish the number of shared species, making it more difficult to identify area-relationships.

In our CADE, the most differentiated area is the Palearctic region, which is the area with the smallest number of earwig clades. Thus, the Palearctic region could be biased towards a basal position in CADE analysis because of its paucity of taxa. The high incidence of endemism can explain, on the other hand, the position of South America (26% of considered clades are endemic to this region, and hence are cladistically uninformative; and a further 21% are uninformative because widespread).

Thus, the basal position of North and South America in CADE is more a reflection of their smaller/endemic faunas and biotic interchanges than a sign of an early split of North and South America from the rest of Gondwana, as South America was separated from Africa later than the separation of Africa from India and Australia [30,80,81,82]. This is also indicated by the fact that the most ancient earwig groups are absent from these latter areas. In general, the increased number of multiple colonization events for Africa and Australia under the DELTRAN assumptions appears in contrast with their history, which is better compatible with the results obtained with the ACCTRAN optimization. The family Karschiellidae, which are considered the most basal lineage of existing Dermaptera, is restricted to tropical Africa, where they probably remained isolated after the Gondwanan break up. The Apachyidae, which are also a very basal clade, are distributed in Africa, Madagascar, India, South East Asia, and Australia (marginally); their absence in South and North America suggests that that this group evolved in Africa and Asia without colonizing southern America when it was still part of the Gondwana. The absence of these groups in North America and the Palearctic region suggests that these areas were colonized later by extant earwigs, as already supposed by Popham [7].

The strong similarity between North and South America can be explained by the biotic interchanges between these two areas occurred as, in the late Cenozoic, the volcanic Isthmus of Panama rose up from the sea floor and bridged North and South America [30,81]. A high incidence of dispersal in the origin of the North American fauna is suggested by the low ci values of the apomorphic distribution supporting this branch in CADE. In particular, an examination of distributional patterns suggests a colonization of North America from South America, as most of the species occurring in North America have their ranges (or belong to lines with ranges) centered in South America (see Popham [7]). In North America, 28 species are known from the USA, and only 7 occur in Canada, whereas about 150 species are known from Brazil (the richest south American country) [4]. Probably, dispersion from South America towards the north was hampered by earwig ecological preferences for hot and humid climates.

The position of Australia in CADE reflects the isolation of this landmass from Africa + Madagascar + India [80,81,82], which is also reflected by the impressive cladogenesis that occurred in this area [33]. However, India was recovered as sister to South East Asia in CADE and was linked to the Palearctic region with the Simpson coefficient, which suggests a biotic interchange occurred after the Indian collision with the Eurasian plate. This biotic interchange seems to have occurred mainly through a dispersal route from India to South East Asia (according to Popham’s [7] reconstruction), whereas the Palearctic Asia was less massively invaded, which can be explained by the barrier represented by the concomitant Himalayan orogenesis and climatic constraints due to earwig preference for hot and humid climates [4]. The position of Madagascar in CADE is likely a reflection of the small number of informative clades. The paleographic history of Madagascar has been long uncertain. Former reconstructions assumed that Madagascar remained close to Africa while India moved northwards [73,74]. However, it is now assumed that Madagascar remained attached to India, when the Somali Basin was opened 121 Myr [80,81,82]. However, in CADE reconstruction, Madagascar is the sister to Africa + (India, South East Asia). This suggests that Madagascar retains a historical signal of its connection with both India and Africa. Use of Dice-Sørensen coefficient suggests a relationship between Madagascar and Australia, and Madagascar appeared to be linked to Australia also in an analysis dealing with scarabaeine dung beetles [83]. However, this may be the reflection of the high incidence of zero values and this relationship was not recovered in the consensus of our cluster analysis.

The overall scenario emerging from the use of βsim, which highlights the pure turnover component, clearly revealed the influence of dispersal events. South East Asia and New Guinea are grouped together, and (at lower similarity) with Australia, which contrasts with the history of Pangea’s fragmentation, but can be explained assuming a dispersal route from Asia to Australia via New Guinea, as hypothesized by Popham [7]. A high incidence of dispersal in the origin of the Australian fauna is also suggested by the low ci values of the apomorphic distributions supporting this branch in CADE. Additionally, India resulted similar to the Palearctic region, which suggests an exchange after the collision of the India subcontinent with the Palearctic Asia about 50 Myr [84]. This is in accordance with the hypothesis of Popham [7] that Southern Palearctic areas such as Tibet, China and Japan were invaded from south of the Himalayas through the deep river valleys of South East Asia.

On the whole, the distribution of major lineages indicates that, in most cases, their evolution predated the Gondwanan fragmentation, which is in accordance with the supposed divergence of the major clades of extant earwigs between about 180 and about 140 Myr [3]. Not only are Karschiellidae limited to tropical Africa and Apachyidae absent from South America, but also the much more advanced Chelisochidae are distributed in Africa, Madagascar, India, South East Asia and Australia (marginally).

The distribution of the genus *Haplodiplatys* in South America, Africa, Madagascar, India and South East Asia may be a reflection of Gondwanan break up. “Pygidicranidae” are a probably paraphyletic group, because of the exclusion of Diplatyidae and are not useful to historical reconstructions. The “Pygidicranidae” are distributed in South America, Africa, India, South East Asia and Australia (marginally). Their radiation was probably influenced by the Cretaceous opening of the Atlantic Ocean, which caused the separation of Diplatyidae Cylindrogastrinae (distributed in South America) and Diplatyidae Diplatyinae (which occur in Africa and India and South East Asia). The “Labiduridae”, which are distributed in all geographic regions, and the “Spongiphoridae”, which are distributed through the southern hemisphere, are probably polyphyletic groups, so their distribution cannot be used to drive historical biogeographic inferences. Anisolabiidae and Forficulidae are distributed worldwide, but some subfamilies have disjunct distributions (e.g., [7]) which can be associated with Gondwanan fragmentation.

Overall, the historical reconstructions of earwig biogeography presented in this paper suggest that these insects evolved on Gondwana and that the colonization of the northern hemisphere was largely hampered by both geographical events (such as the Himalayan orogenesis, which hindered earwig dispersal into the Palearctic region) and climatic factors (because of earwig preferences for warm and humid climates). This reconstruction may provide an explanation for the decline of earwig diversity from the equator to the poles within the so-called tropical conservatism hypothesis, which postulates that the current high diversity in the tropics results from the tropical origins of extant clades, a longer time for cladogenesis in tropical environments because of their environmental stability, and the reduced capabilities of organisms from historically tropical lineages to adapt to temperate climates [4].

## 5. Conclusions

Our analyses indicate that the major patterns in earwig global distribution were determined by vicariance events (led by the break-up of Gondwana) followed by large scale dispersal processes constrained by the Himalayan orogenesis and presence of colder temperatures, which have largely hindered the colonization of the northern hemisphere. We are aware of the limits of our study, which are (1) the use of very broad areas of endemism; (2) the use of terminal taxa of different taxonomic levels; and (3) the lack of a fully resolved phylogeny for all considered taxa. However, we think that our results are sufficiently solid as the use of broad regions and high taxonomic levels (subfamilies and genera) avoid uncertainties due to the imperfectly known distributions at species or genus level at a finer scale. Additionally, for the purpose of CADE, what is important is monophyly, not taxonomic rank, and use of equal lower ranks (e.g., species or genera in all cases) would only increase, in most cases, the number of apomorphic, uninformative distributions. Finally, although we were unable to specify the phylogenetic relationships of all terminal clades, all groups introduced in CADE were monophyletic to the best of our knowledge. This means that we might have less support than possible, but area-relationships should be not influenced. Our exercise has provided testable hypotheses and we hope that future works, based on refined data (e.g., molecular reconstructions), will test them.

## Data Availability

Data are given in Tables S1 and S2.

## Supplementary Materials

The following supporting information can be downloaded at: https://www.mdpi.com/article/10.3390/biology11121794/s1, Figure S1: Cladistic relationships among earwigs used in this study. The cladogram is based on the phylogenomic analysis of Wiplfer et al. [37]. Clades in grey were not included in the phylogenomic dataset of Wiplfer et al. [37], and their relationships were based on morphological (Karskiellidae) and molecular and morphological (Arixeniidae) analyses presented in other studies reviewed in Wiplfer et al. [37]; Table S1: Taxon distribution across areas of endemism; Table S2: Matrix used for CADE; Table S3: Character diagnostics for Figure 2. Consistency index (ci), retention index (ri), rescaled consistency index (rc) and homoplasy index (hi) are presented for each “character” (taxon); Table S4: Apomorphy list for Figure 2. A double-lined arrow means that the change occurs in all possible reconstructions (i.e., is unambiguous); characters with such changes are uniquely derived synapomorphies. A single line arrow indicates that changes occur under some reconstructions but not others. Character state changes are shown under ACCTRAN and DELTRAN optimization.
