# Supplementary figures and images for: Historical Biogeography of Earwigs
Source: Biology (Basel). 2022 Dec 9;11(12):1794. doi: 10.3390/biology11121794 (PMC9775502; doi:10.3390/biology11121794)

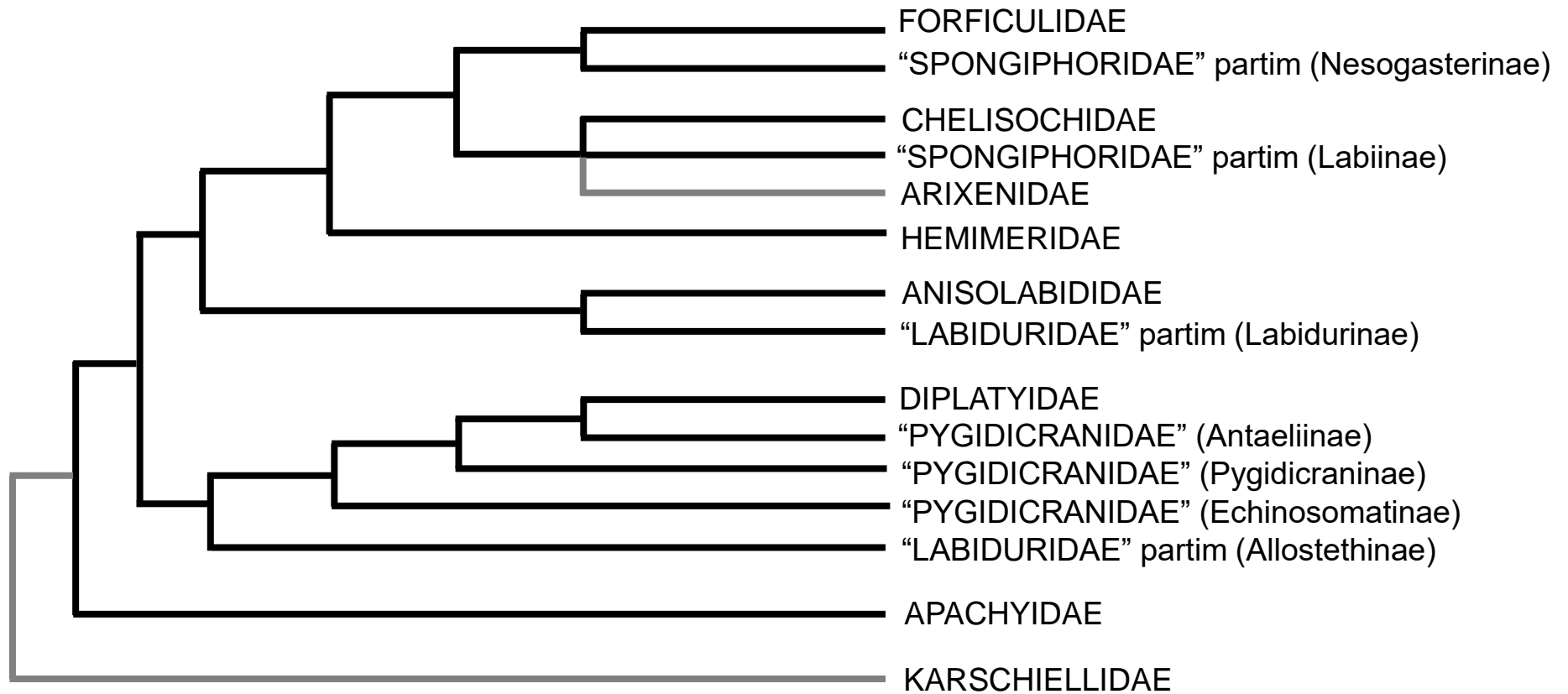

Supplement: Supplementary file 1 [file biology-11-01794-s001.zip › Dermaptera_Supplementary/Figure S1.pdf]
